# Conservative Surgery in Endometrial Cancer

**DOI:** 10.3390/jcm11010183

**Published:** 2021-12-29

**Authors:** Alessandra Gallo, Ursula Catena, Gabriele Saccone, Attilio Di Spiezio Sardo

**Affiliations:** 1 Department of Public Health, School of Medicine, University of Naples Federico II, 80131 Naples, Italy; attiliodispiezio@libero.it; 2 Division of Gynecologic Oncology, Fondazione Policlinico Universitario Agostino Gemelli IRCCS, 00168 Rome, Italy; ursula.catena@gmail.com; 3 Department of Neuroscience, Reproductive Sciences, and Dentistry, School of Medicine, University of Naples Federico II, 80131 Naples, Italy; gabriele.saccone.1990@gmail.com

**Keywords:** endometrial cancer, fertility-sparing, hysteroscopy, progestins

## Abstract

Endometrial cancer (EC) is the sixth most common female cancer worldwide. The median age of diagnosis is 65 years. However, 4% of women diagnosed with EC are younger than 40 years old, and 70% of these women are nulliparous. These data highlight the importance of preserving fertility in these patients, at a time when the average age of the first pregnancy is significantly delayed and is now firmly established at over 30 years of age. National Comprehensive Cancer Network (NCCN guidelines state that the primary treatment of endometrial endometrioid carcinoma, limited to the uterus, is a total hysterectomy, bilateral salpingo-oophorectomy and surgical staging. Fertility-sparing treatment is not the standard of care, and patients eligible for this treatment always have to undergo strict counselling. Nowadays, a combined approach consisting of hysteroscopic resection, followed by oral or intrauterine-released progestins, has been reported to be an effective fertility-sparing option. Hysteroscopic resection followed by progestins achieved a complete response rate of 95.3% with a recurrence rate of 14.1%. The pregnancy rate in women undergoing fertility-sparing treatment is 47.8%, but rises to 93.3% when only considering women who tried to conceive during the study period. The aim of the present review is to provide a literature overview reflecting the current state of fertility-sparing options for the management of EC, specific criteria for considering such options, their limits, the implications for reproductive outcomes and the latest research trends in this direction.

## 1. Introduction

Endometrial cancer (EC) is the sixth most common female cancer worldwide, with approximately 417,000 new cases and 97,000 deaths in 2020 [1]. The median age of diagnosis of EC is 65 years, as it occurs mostly in postmenopausal women. However, 4% of women diagnosed with EC are younger than 40 years old; 70% of these women are nulliparous [2] or women whose reproductive desire has not yet been fulfilled at the time of diagnosis. These young patients (40 years old or less) have excellent 5-year survival rates, over 95%, as these women are more likely to present with endometrioid, focal, well-differentiated tumors, limited to the endometrium or superficial myometrium [3]; in other words, 80% of cases are early EC, stage IA, according to the International Federation of Gynecology and Obstetrics [FIGO] staging system. These data highlight the importance of preserving fertility in these women, at a time when the average age of the first pregnancy is significantly delayed and is now firmly established at over 30 years of age.

## 2. Objectives

The aim of the present review is to provide a literature overview reflecting the current state of fertility-sparing options for the management of EC, specific criteria for considering such options, their limits, the implications for reproductive outcomes and the latest research trends in this direction. In particular, the following topics were evaluated and analyzed:

- Risk factors for endometrial hyperplasia and cancer
- Screening and diagnosis
- Treatment
- Follow-up
- Oncological outcomes
- Reproductive outcomes

### 2.1. Risk Factors for Endometrial Hyperplasia and Cancer

EC develops as the evolution of endometrial hyperplasia, a cancer precursor lesion that is differentiated into two categories, according to the latest classification system of the World Health Organization (WHO), which in 2014 developed an update of the old system, classifying these lesions into “non-atypical (benign) hyperplasia” and “atypical hyperplasia or endometrioid intraepithelial neoplasia (EIN)”. The main risk factor for these precursor lesions and for EC itself is the prolonged—unopposed by progesterone antagonization—estrogen exposure [4]. Several conditions such as nulliparity, chronic anovulation, irregular menstrual cycles and non-insulin-dependent diabetes are associated with a higher risk of developing EC particularly in young women [5,6]. The association between the risk of EC and PCOS, which is characterized by most of the conditions listed above, has been suggested and demonstrated for many years [7].

Women with metabolic syndrome have a relative risk (RR) of 1.89 of developing EC [8]. Obesity is an independent risk factor, and it can be related to 40–50% of EC cases; this association is found to be greater in postmenopausal women compared with women of reproductive age [9]. The strength of this association increases, increasing BMI, with an RR of 1.32 for overweight women and 2.54 for obese women [10]. In young women, the effect of obesity on endometrial cancer has only been found when the obesity is severe or morbid [9]. In addition, BMI affects the response to treatment, with a relapse rate of 32.6% for women with a BMI of 35 or more, conservatively treated with a levonorgestrel-intrauterine system (LNG-IUS), versus 3.3% for women with a BMI of less than 35 [11].

These data highlight that a balanced diet, healthy lifestyle and physical activity are important for the prevention of EC, as strongly recommended by the World Cancer Research Fund back in 2007 [12].

In addition, genetic predisposition to EC exists, as a part of Lynch syndrome, caused by germline mutations in DNA mismatch repair genes, with autosomal dominant inheritance. In women with this syndrome, the risk of developing endometrial cancer is about 20–30% [13], but it significantly increases when it is concomitantly diagnosed with type-2 diabetes and metabolic syndrome [14,15]. Lastly, exposure to tamoxifen for breast cancer therapy shows a risk of EC 2.53 times higher than that for women of the same age who have not been exposed, and the level of risk is affected by dose and time of the treatment [16,17].

### 2.2. Screening and Diagnosis

There is no evidence for efficacy of EC screening among women without any risk factors or symptoms. Many studies show that screening of asymptomatic women by ultrasound does not affect the mortality rates of EC, and rather results in unnecessary second level exams due to false-positive results [18].

In contrast, women diagnosed with Lynch syndrome should be offered an annual ultrasound and endometrial biopsy starting at the age of 35, to be performed preferably with a minimally invasive approach, up until the prophylactic hysterectomy and bilateral salpingo-oophorectomy, as this is carried out from the age of 40 [19]. On the other hand, detection of EC in a patient younger than 50 years old should be considered as a sentinel event for Lynch syndrome, and a diagnostic pathway for suspected hereditary cancer should be undertaken [20].

The clinical presentation of EC in postmenopausal women consists primarily of abnormal uterine bleeding (AUB), which must always be investigated. In premenopausal women, symptoms may also include AUB (heavy menstrual bleeding, disorders of frequency, duration or regularity of menstruation) but also intermenstrual bleeding, which a recent systematic review has associated with a higher risk than the other symptoms [21].

A transvaginal ultrasound should be used as a first-line diagnostic tool, to select patients for second level evaluation; the cornerstone examination for the diagnosis of EC consists of an endometrial biopsy. Several methods to collect endometrial tissue samples exist, such as Pipelle, Novak curette, Vabra^®^, blind Dilation and Curettage (D&C) or hysteroscopic guided endometrial biopsy [22]. D&C has long been considered the elective method to obtain histology, and it is still preferred by many authors [23], despite its many deficiencies. Falcone et al. in 2017 showed that D&C is associated with the lowest rate (<10%) of histological under-grading [24], and it has been proven to be superior, compared with the Pipelle biopsy, in terms of correlation with the histological result of the final specimen [25]. Several studies disagree and conclude that, as it is performed with a blind approach, it is often able to sample less than 50% of the endometrial cavity; consequently, nearly 10% of endometrial lesions could be missed, particularly if they are focal, with a high percentage of false-negative results [26,27]. Ramshaw et al. suggest that blind techniques should no longer be offered to obtain endometrial histology [28], favoring instead the hysteroscopic method, which is increasingly used for the diagnosis of EC.

Performed in an office setting and allowing direct visualization of the uterine cavity, the hysteroscopic guided “grasp” endometrial biopsy, using 5 Fr instruments, is the gold-standard in the diagnosis of endometrial malignancy. It achieves high concordance of histologic type and tumor grade, especially in the presence of an endometrioid-type tumor [22] and allows the distinction between endocervical mucosal infiltration and an EC protrusion into the endocervical canal; therefore, it enables the obtention of a preoperative diagnosis of EC and helps to inform the decision on therapeutic management.

#### Imaging

Pelvic magnetic resonance imaging (MRI) should be performed to exclude myoinvasion and assess local disease extent; a pelvic transvaginal (TV) ultrasound should be performed if MRI is contraindicated [29]. MRI demonstrated a high diagnostic accuracy in terms of myometrial infiltration, cervical invasion and lymph node metastases [30].

According to some authors, TV ultrasound shows a good level of accuracy in the local staging of endometrial carcinoma when carried out by expert practitioners. Because of its high costs, MRI should be offered only to those patients for whom TV ultrasound produces images of poor quality [31,32,33].

### 2.3. Treatment

The most recent NCCN guidelines state that the primary treatment of endometrial endometrioid carcinoma, limited to the uterus, is a total hysterectomy, bilateral salpingo-oophorectomy and surgical staging [29], preferably with a minimally invasive approach when technically feasible; even young women interested in future fertility, who may benefit from fertility-sparing options, should always undergo counselling to inform them that this option is not the standard of care. In fact, they should also be encouraged to conceive as soon as the complete response is achieved and, after childbearing is completed, to then undergo radical surgery.

All of the following criteria for considering fertility-sparing options for the management of endometrial carcinoma must be met:

1. The patient must be diagnosed with well-differentiated (Grade 1) endometrioid adenocarcinoma on D&C, confirmed by expert pathology review.
2. The disease must be limited to the endometrium on MRI (preferred) or TV ultrasound.
3. There must be an absence of suspicious or metastatic disease on imaging.
4. There must be no contraindications to medical treatment or pregnancy.

Malignant carcinoma other than pure endometrioid carcinoma, such as serous carcinoma, clear cell carcinoma, undifferentiated carcinoma, and choriocarcinoma, as well as malignant mesenchymal (sarcoma), should be treated as high-grade endometrial cancer and therefore fertility-sparing surgery is not recommended [29].

In addition, some experts have also proposed adding the presence of strong and diffuse expression of progesterone receptor (PR) among the criteria on the immunohistochemistry (IHC) staining [33].

The cornerstone of the fertility-sparing treatment for EC and its precursor AEH has traditionally been continuous progestin-based therapy. According to the most recent published guidelines, orally administered megestrol acetate (160–320 mg/day) or medroxyprogesterone acetate (400–600 mg/day) is the recommended treatment. Local treatment with levonorgestrel-releasing intrauterine system (LNG-IUS), in combination with oral progestins with or without gonadotropin-releasing hormone analogs can also be considered [33,34,35,36]. More recently, a combined approach consisting of hysteroscopic resection, followed by oral or intrauterine-released progestins, has been reported to be an effective fertility-sparing treatment [24,37,38,39].

Mazzon et al. first described the “three steps” hysteroscopic technique, consisting of a resection of the tumor lesion (Step 1), the endometrium adjacent to the lesion (4–5 mm outside) (Step 2) and the myometrium underlying the lesion (3–4 mm) (step 3); once the pathology report confirmed Grade 1 (G1) EC without myometrial invasion, then medical therapy with megestrol acetate (160 mg daily) was administered for 6 months [40].

Giampaolino et al. described a combined fertility-sparing treatment, but they made a distinction between early endometrial carcinoma (EEC) and AEH. Patients diagnosed with EEC underwent hysteroscopic resection following the “three steps technique” by Mazzon, adding multiple random endometrial biopsies; an LNG-IUD was inserted when the histologic report confirmed EEC G1 on the lesion, with the surrounding endometrium and the underlying myometrium free of disease. In cases where AEH is diagnosed, the surgical treatment consisted of superficial endometrial resection, preserving the basal layer of the endometrium, followed by LNG-IUS insertion right after the procedure [38].

If we consider endometrial cancer on an endometrial polyp, we can find that De Rijk et al. showed an estimated risk of 5.6% (95% CI 0.2–17.6%) on concurrent endometrial cancer when atypia is found within an endometrial polyp [41]. The incidence of endometrial carcinoma in the surrounding endometrium after complete resection of a polyp with atypical hyperplasia is 30.8% in a more recent study [42]. This supports the advice not to change the management in this case.

### 2.4. Follow-Up

There is still no unified consensus on follow-up strategies, especially for the frequency and method of endometrial sampling and the time to perform definitive radical surgery. Instead, there is a unanimous agreement on the critical importance of patient compliance to accept the need for careful follow-up [43].

According to NCCN guidelines, endometrial evaluation should be performed every 3–6 months, either with D&C or an endometrial biopsy [29]. Canadian Clinical Practice Guidelines point out that endometrial surveillance should be ensured every 3 months, until at least two negative specimens are obtained [25]; follow-up should continue with endometrial biopsy every 6 months for 2 years, and then every year, until definitive surgery is performed. Falcone et al. suggest that follow-up should include general and gynecological examinations, transvaginal ultrasonography (TVS), serum cancer antigen 125 (CA-125) and diagnostic hysteroscopy every 3 months, then an abdomen-pelvis computed tomography (CT) at 6 months and 6-monthly thereafter [24]. Giampaolino et al., in their internal protocol, established that the LNG-IUS had to be maintained in situ for at least 12 months; women with CR on the last two biopsies removed the LNG-IUS and tried to conceive [38]. Koskas et al., in their meta-analysis, showed that there is no statistically relevant difference between the remission probability after 6 months or 12 months of treatment [44].

In summary, the most established and reasonable option for the surveillance seems to be a hysteroscopic endometrial biopsy at 3 and 6 months; then, if the patient is not ready to conceive immediately, progestin therapy should be offered and follow-up should continue every 6 months for two years, deferring it every year thereafter. Patients who achieve a complete response (CR) by 6 months, and wish to conceive, should be advised to actively pursue it, and should consider assisted reproductive technologies (ART) to improve success rates, with continued surveillance by endometrial sampling every 6 months. No maximum time frame within which to conceive is reported in literature; however, higher pregnancy and live birth rates are reported for women who used ART, compared with spontaneous conception attempts [45].

Definitive surgery should be performed after completion of pregnancy or if there is evidence of disease progression at endometrial sampling. In cases of recurrence or non-response at 6–12 months follow-up, the gynecologic oncology societies suggest a total hysterectomy with bilateral salpingo-oophorectomy; the same in cases of progression to carcinoma from hyperplasia or decline in endometrial surveillance. A second course of conservative treatment (medical therapy or combined treatment) could be performed in cases of refusal of definitive surgical treatment or in patients with high surgical risk [25,43].

### 2.5. Outcomes

Table 1 shows the summary of our main findings about oncological and reproductive outcomes.

#### 2.5.1. Oncological Outcomes

CR rates correlate with the expression of hormone receptors on neoplastic cells; they range from 26% to 89% in estrogen and progesterone receptor-positive tumors, whereas they fall to 8–17% in receptor-negative tumors [46].

A recent review and meta-analysis, including 28 studies and 1038 women, showed that women diagnosed with early endometrial cancer (EEC) or AEH who underwent conservative management with oral progestins had a pooled CR rate of 71% (95% confidence interval [CI]: 63–77%). The pooled CR for women using LNG-IUS was 76% (95% CI: 67–83%), with a pooled recurrence rate (RR) of 9% (95% CI: 5–17%). Patients using oral progestin plus LNG-IUS showed a pooled CR rate of 87% (95% CI: 75–93%) [47]. However, in this case, no distinction is made between EEC and AEH.

Gallos et al. made that distinction in a meta-analysis, reporting a pooled CR of 76.2% for EEC and 85.6% for AEH [45] although no separate rates for each kind of progestin treatment were reported. The same group had earlier showed LNG-IUS to have more effectiveness than oral progestins in women affected by AEH [48].

A previous review by Ramirez et al. reported 27 articles containing 81 patients with EEC, treated with oral progestin for 3 months, showing a CR rate of 76%, with a mean response time of 12 weeks. Among them, the RR was 24%, in over a mean of 19 weeks, but this review did not address the use of LNG-IUS as a means of treatment [35]. In fact, the use of LNG-IUS in women with a diagnosis of EEC has not been widely reviewed as well as the use of oral progestins.

In a meta-analysis, Fan et al. reported a CR rate of 95.3% in cases of hysteroscopic resection followed by progestins, compared with 76.3% and 72.9% in cases of oral progestins alone or LNG-IUS alone, respectively. The RR was 14.1% in cases of hysteroscopic resection plus progestins, compared with 30.7% and 11% in cases of oral progestins alone or LNG-IUS alone, respectively [49].

Giampaolino et al. reported that the combination of hysteroscopic resection followed by LNG-IUS insertion achieves similar response rates and considerably lower RR compared with those reported in literature for progestins alone. Of the 14 patients diagnosed with EEC, 11 (78.6%) achieved a CR, two (18.2%) of whom had subsequent relapse; of the 55 patients diagnosed with AEH, 51 (92.7%) achieved a CR, two (3.9%) of whom had subsequent relapse [38].

Unfortunately, a variable percentage of patients shows unfavorable outcomes, such as no regression of the disease or a recurrence or progression to more advanced disease [47]. The recurrence rate of EC, including both local and systemic recurrence, after conservative treatment is between 20–35% in 4 to 66 months, according to different studies, and is therefore higher compared to standard surgical treatment.

For this reason, in the last few years, the search for predictive markers of response to conservative treatment appears crucial, but no marker has shown a predictive accuracy so high that it can be used as a stand-alone marker [50].

Recent studies have expanded our understanding of the genomic features of EC, leading to the identification of molecular features predictive of individual tumor behavior. The Cancer Genome Atlas (TCGA) project stratified EC into four genomically defined prognostic subgroups: POLE ultramutated, microsatellite instability hypermutated, copy number low and copy number high. POLE-mutant tumors have significantly better progression-free survival, whereas copy-number high tumors have the poorest [51].

In the last few years, using low cost and simple assays broadly available in clinical practice, surrogate markers have become available to easily determine these four subgroups. In particular, the Proactive Molecular Risk Classifier for Endometrial cancers (ProMisE), which is based on three key components: immunohistochemistry (IHC) for the presence of mismatch repair (MMR) proteins, sequencing for the presence of POLE exonuclease domain mutations (EDMs) and IHC for p53 [52].

It has been reported that this molecular classifier model works successfully on endometrial biopsies or curettages with high concordance with the hysterectomy specimens [53]. In a fertility-sparing cohort of patients, this model could be a very useful option for an integrated classification system allowing a more reliable EC prognostic evaluation and early hereditary cancer risk assessment.

In 2019, Falcone et al. proposed to test the ProMisE in patients conservatively treated for EC, demonstrating the feasibility of a molecular categorization in a fertility-sparing setting [54].

Moreover, resistance to conservative treatment is demonstrated to be more common in MMR-deficient patients than in MMR-proficient patients (33.3% vs. 15.9%; RR = 2.1), but with no statistical significance. Recurrence is significantly more common in MMR-deficient patients than in MMR-proficient cases (100% vs. 26.4%; RR = 3.8; *p* < 0.0001). In these patients, a closer and more careful follow-up may be necessary given the higher risk of recurrence [55].

#### 2.5.2. Reproductive Outcomes

A meta-analysis of 34 articles and 559 women, 408 with EEC and 151 with AEH, managed with fertility-sparing treatment, found encouraging live birth rates of 28% and 26.3%, respectively. It also showed better results for women who choose ART [47]. Gallos et al. found that the difference between assisted reproduction and spontaneous conception in achieving a live birth was statistically significant, with a live birth rate of 39.4% in women who used ART, compared with 15% in women who tried to conceive spontaneously [45]. The type of ovarian stimulation and the ART protocol should be tailored on the basis of the characteristics of each patient, in consultation with a multidisciplinary team.

More recently, analysis of 1038 patients with EEC and AEH showed worse reproductive outcomes for patients treated with LNG-IUS alone, compared with patients treated with oral progestin, with or without LNG-IUS. A total of 34% of women taking oral progestins became pregnant, but only 20% delivered live newborns; pregnancy rates for women taking LNG-IUS was 18%, and 14% delivered live newborns; 40% of women taking oral progestins plus LNG-IUS had a pregnancy, and 35% delivered live newborns [47]. Fan et al., showed instead higher pregnancy rates in women taking LNG-IUS, 56% vs. 52.1% in women taking oral progestins alone and 47.8% for combined treatment with hysteroscopic resection followed by progestin therapy [49].

Combined treatment was also prospectively evaluated by Falcone et al., who reported a pregnancy rate of 53.8% and a live birth rate of 50%. Pregnancy rates and live birth rates, however, rose to 93.3% and 86.6%, respectively, when only considering women who tried to conceive during the study period, which appear higher than those reported for medical therapy alone [24].

Giampaolino et al., in their series, achieved a pooled live birth rate of 14.5% which rose up to 26.3%, in the range previously reported, when excluding women who had not attempted to become pregnant in the short term. No patient with EEC became pregnant, while 18.2% patient with AEH achieved pregnancy and live birth; a hypothesis is that the short follow-up time could have affected the rates, but in most series, pregnancies primarily occur in the first two years [38]. The possibility that the hysteroscopic resection technique performed in cases of EEC could damage the basal layer of the endometrium, affecting reproductive chances, has been theorized and should be evaluated.

However, these overall finding suggest that the combined treatment could be considered a safe approach in the management of EEC and AEH, that does not affect reproductive outcomes.

## 3. Discussion

### 3.1. Main Findings

**Figure 1:**
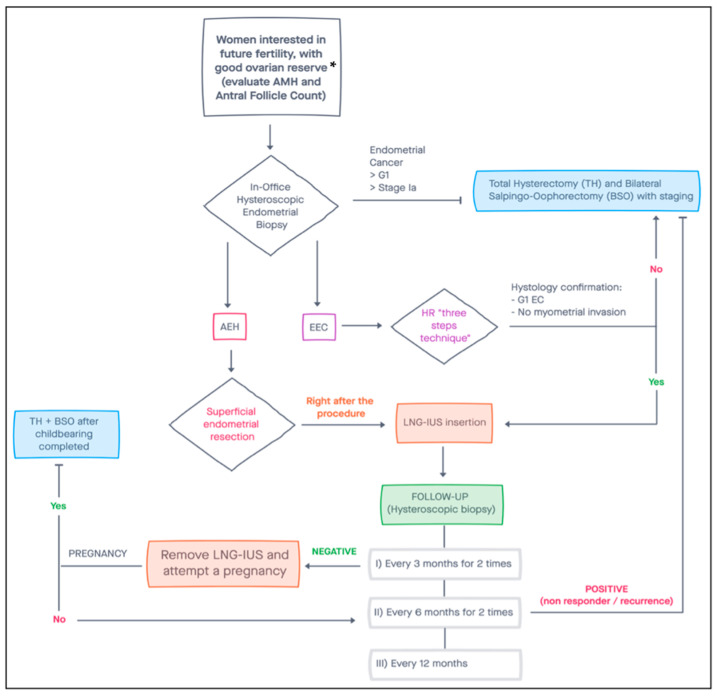
Suggested flow-chart for conservative management of women with endometrial cancer. * Patients with a diminished ovarian reserve may still benefit from fertility-sparing surgery, attempting a pregnancy with heterologous oocytes. AMH, Anti-Müllerian Hormone; AEH, Atypical Endometrial Hyperplasia; EEC, Early Endometrial Carcinoma; EC, Endometrial Cancer; HR, Hysteroscopic Resection; LNG-IUS, Levonorgestrel Intrauterine System.

This study aimed to review all the evidence on conservative treatment of EC, including risk factors, screening and diagnosis, imaging, treatment, follow-up and oncological outcomes. We also suggest a flow-chart to manage EC with a conservative approach, based on findings of this review (Figure 1).

### 3.2. Implication and Future Perspective

As mentioned above, well-differentiated (Grade 1) endometrioid adenocarcinoma and disease limited to the endometrium are among criteria for considering fertility-sparing options. However, some evidence is reported in literature about the conservative treatment of early-stage G2 endometrioid adenocarcinoma or well-differentiated G1 endometrioid adenocarcinoma with minimal myometrial invasion, but they are limited mostly due to the exclusion of these cases from fertility-sparing programs. Casadio et al. described encouraging oncological and reproductive outcomes of conservative fertility-sparing management for women diagnosed with a well-differentiated G1 endometrial endometrioid adenocarcinoma, with minimal myometrial infiltration (1–2 mm), free resection margins and the absence of lymphovascular invasion [3,56]. Performing a combined treatment, with hysteroscopic resection according to Mazzon’s technique, followed by hormone therapy (megestrol acetate 160 mg daily) for 9 months, CR was achieved, offering a chance to women who have not yet fulfilled their desire to have children. Recently, a multicenter study proposed conservative treatment by progestins, with or without hysteroscopic resection, for patients affected by moderately differentiated endometrioid-type adenocarcinoma (grading G2) [57]. In a follow-up period of about 3 years, a CR rate of 73.9% was observed, with a recurrence rate of 41.1%, which is consistent with previous studies (71.4%) [58,59,60,61], and not so different from rates obtained in G1 patients. Data on reproductive outcomes in this group are inconclusive, with a live birth rate of 17.6% among all complete responders, also considering women who did not actively try to conceive; this low result may be linked to the higher mean age of women included.

## 4. Conclusions

A total of 4% of women diagnosed with EC are younger than 40 years old. The current mean age of the first pregnancy is significantly delayed and is now firmly established at over 30 years of age. These data highlight the importance of preserving fertility in these women.

EC in young patients has more favorable characteristics. However, the fertility sparing approach is considered a non-standard care approach and even young women interested in future fertility, who may benefit from fertility-sparing options, should always undergo counselling informing them that this option is not the standard of care.

Hysteroscopic resection followed by progestins achieved a CR rate of 95.3%, compared to 76.3% and 72.9% in cases of treatment with oral progestins alone or LNG-IUS alone, respectively [49]. Moreover, a combined hormonal treatment with medroxyprogesterone acetate given orally and LNG-IUS reached a CR rate of 87.5% [62].

The RR was described as 14.1% in cases of hysteroscopic resection plus progestins, compared with 30.7% and 11% in cases of oral progestins alone or LNG-IUS alone respectively [49]. Pregnancy rates in women undergoing hysteroscopic resection followed by progestin therapy was 47.8%, compared with 52.1% and 56% in cases of treatment with oral progestins alone or LNG-IUS alone, respectively. Pregnancy rates and live birth rates rise to 93.3% and 86.6%, respectively, when only considering women who tried to conceive during the study period [24].

However, a variable percentage of patients shows unfavorable outcomes and the research of predictive markers of response to conservative treatment appears crucial in recent years. The Proactive Molecular Risk Classifier for Endometrial cancers (ProMisE) is demonstrated to be a low-cost and simple tool, broadly available in clinical practice, to easily determine the patients risk subgroup; it is based on three key components: IHC for MMR proteins, sequencing for the presence of POLE EDMs and IHC for p53 [52].

This molecular classifier model works successfully on endometrial samplings with high concordance with the final hysterectomy specimens [53]. So, in a fertility-sparing cohort of patients, this model could be a very useful option for an integrated classification system allowing a more reliable EC prognostic evaluation and early hereditary cancer risk assessment.

Resistance to conservative treatment is more common in MMR-deficient patients than in MMR-proficient patients (33.3% vs. 15.9%; RR = 2.1), but with no statistical significance. Recurrence is significantly more common in MMR-deficient patients than in MMR-proficient cases (100% vs. 26.4%; RR = 3.8; *p* < 0.0001). In these patients, a closer and more careful follow-up may be necessary given the higher risk of recurrence [55].

Finally, hysteroscopic resection in combination with progestin therapy is significantly associated with shorter treatment duration to achieve CR and a longer time to relapse than treatment with progestins alone. Available literature provides convincing data on the efficacy of this approach compared with the conservative treatment of patients with EEC and AEH, but prospective randomized multi-institutional trials comparing the efficacy of different progestin treatments are needed in the future to better define the risk assessment on the base of a molecular approach.

## Figures and Tables

**Table 1:** Oncological and Reproductive outcomes of fertility-sparing treatment of endometrial cancer.

First Author and Year	N. of Patient	Histology	Type of Treatment	Complete Response Rate	Recurrence Rate	Pregnancy Rate	Live Birth Rate
Ramirez 2004	81	EEC	OP	76%	24%	N.A.	N.A.
Gallos 2012	559	408 EEC 151 AEH	N.A.	76.2% 85.6%	40.6% 26%	N.A. N.A.	28% 26.3%
Falcone 2017	28	EEC	HR + OP/HR + LNG-IUS	96.3%	7.7%	93.3% ^1^	86.6% ^1^
Fan 2017	619	EEC	HR + OP OP LNG-IUS	95.3% 76.3% 72.9%	14.1% 30.7% 11%	47.8% 52.1% 56%	N.A N.A. N.A.
Wei 2017	1038	EEC/AEH	OP LNG-IUS OP + LNG-IUS	71% 76% 87%	20% 9% N.A.	34% 18% 40%	20% 14% 35%
Giampaolino 2018	69	14 EEC 55 AEH	HR + LNG-IUS	78.6% 92.7%	18.2% 3.9%	0% 26.3% ^1^	0% 26.3% ^1^

EEC, Early Endometrial Cancer; AEH, Atypical Endometrial Hyperplasia; OP, Oral Progestins; HR, Hysteroscopic Resection; LNG-IUS, Levonorgestrel Intrauterine System. ^1^ Considering only women who tried to conceive.
